# A new method to measure the semantic similarity from query phenotypic abnormalities to diseases based on the human phenotype ontology

**DOI:** 10.1186/s12859-018-2064-y

**Published:** 2018-05-08

**Authors:** Xiaofeng Gong, Jianping Jiang, Zhongqu Duan, Hui Lu

**Affiliations:** Department of Bioinformatics and Biostatistics, SJTU-Yale Joint Center for Biostatistics, Shanghai Jiao Tong University, Shanghai, China

**Keywords:** Human phenotype ontology (HPO), Semantic similarity, Disease, Diagnosis

## Abstract

**Background:**

Although rapid developed sequencing technologies make it possible for genotype data to be used in clinical diagnosis, it is still challenging for clinicians to understand the results of sequencing and make correct judgement based on them. Before this, diagnosis based on clinical features held a leading position. With the establishment of the Human Phenotype Ontology (HPO) and the enrichment of phenotype-disease annotations, there throws much more attention to the improvement of phenotype-based diagnosis.

**Results:**

In this study, we presented a novel method called RelativeBestPair to measure similarity from the query terms to hereditary diseases based on HPO and then rank the candidate diseases. To evaluate the performance, we simulated a set of patients based on 44 complex diseases. Besides, by adding noise or imprecision or both, cases closer to real clinical conditions were generated. Thus, four simulated datasets were used to make comparison among RelativeBestPair and seven existing semantic similarity measures. RelativeBestPair ranked the underlying disease as top 1 on 93.73% of the simulated dataset without noise and imprecision, 93.64% of the simulated dataset with noise and without imprecision, 39.82% of the simulated dataset without noise and with imprecision, and 33.64% of the simulated dataset with both noise and imprecision.

**Conclusion:**

Compared with the seven existing semantic similarity measures, RelativeBestPair showed similar performance in two datasets without imprecision. While RelativeBestPair appeared to be equal to Resnik and better than other six methods in the simulated dataset without noise and with imprecision, it significantly outperformed all other seven methods in the simulated dataset with both noise and imprecision. It can be indicated that RelativeBestPair might be of great help in clinical setting.

## Background

Correct diagnosis based on the observed clinical features of patients is a quite important task for physicians, especially in the field of rare genetic diseases, where different diseases often share some features. Recently, with the rapid development of sequencing technology, it becomes possible to improve diagnosis by providing physicians with patients’ genotype data in a short time [1]. While techniques like whole genome sequencing and whole exome sequencing allows a patient’s genotype data to be used to detect mutations, the relative high expense and the ability to identify disease-causing variants make it difficult to be put into practical clinical use. However, back to the beginning, if the performance of diagnosis based on clinical features can be improved, it will be of great help to the clinicians.

Thus, to make full use of clinical features or phenotypic information, many databases have been established to record and reorganize phenotypic data of diseases, such as OMIM [2] and Orphanet [3]. Furthermore, the Human Phenotype Ontology (HPO) [4–6] was constructed to describe human phenotype abnormalities in a structured and controlled vocabulary and has been widely used in research.

Recently, HPO has been widely applied in various fields. A web application called the *Phenimizer* provides ontology similarity search based on HPO to assist the clinical diagnosis workflow [7]. *PhenoTips*, a deep phenotyping tool and database, is developed to collect phenotypic information of patients with genetic disorders using HPO and suggest additional clinical investigations and possible disorders in Online Mendelian Inheritance in Man (OMIM) [8]. *PhenoDB*, a Web-based portal which can store and analyze phenotypic information using mapped HPO terms as well as other clinical information, is also developed [9]. Besides, several methods or tools have been introduced to combine phenotypic information based on HPO and genotypic data with other information available to make variant or gene prioritization, including eXtasy [10], Phen-Gen [11], an initial study using semantic similarity [12], PHIVE/Exomiser [13], Phevor [14], PhenoVar [15], PhenIx [16] and OMIM Explorer [17]. Despite the short history of HPO, it has drawn much attention from researchers and scientists and been broadly used in scientific researches.

In this article, we focus on using similarity between observed phenotypes of a patient and the annotated phenotypes of diseases to rank the candidate diseases of the patient. From this point of view, several methods and tools [7, 12, 18] has been presented to exploit HPO-based semantic similarity borrowing ideas from semantic similarity measures used in Gene Ontology (GO), which have been widely studied and broadly used during the last decade. Most of them utilized information content (IC) to calculate the semantic similarity. Although those approaches have been used in clinical research, the results are still uncertain and can be further imporved. Here we present a new method called RelativeBestPair. RelativeBestPair takes the ideas from information content and the best pair method. Our work shows better diagnosis using the RelativeBestPair method over other methods.

## Methods

### Human phenotype ontology (HPO)

**Fig. 1:**
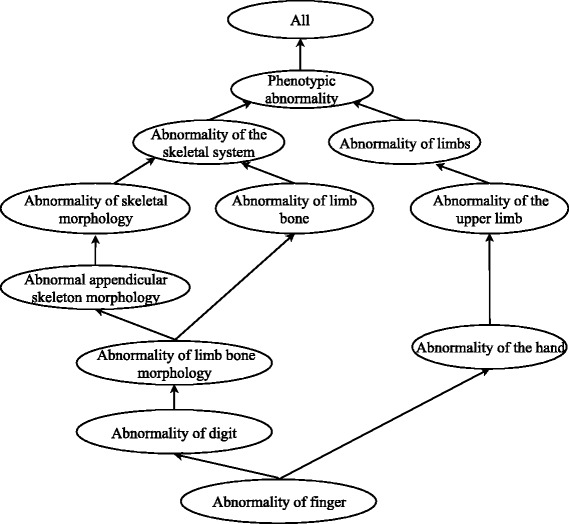
Example of the structure of HPO. Term Abnormality of finger (HP:0001167) and all its ancestors are shown. Each term, representing a phenotypic abnormality, is related to parents terms by “is a” relationship

An ontology is a knowledge-based structured system, which consists of a rich, standardized vocabulary to describe entities and the semantic relationships between them. The Human Phenotype Ontology (HPO) provides a standardized vocabulary of phenotypic abnormalities encountered in human disease. Terms in HPO, representing different phenotypic abnormalities, are related to their parent terms by “is a” relationship in a relaxed hierarchy which allows a term to possibly have multiple parent terms (Fig. 1). With HPO terms corresponding to phenotypic abnormalities, diseases can be described in a detailed and organized way. The HPO (version 1.2 releases/2017–2-14) currently contains approximately 12,000 terms (still growing) and over 120,000 phenotype-disease annotations. Here we concentrate on annotations about 6918 diseases listed in Online Mendelian Inheritance in Man (OMIM) to calculate the semantic similarity scores.

### RelativeBestPair method

Based on the HPO structure and annotations, the information content of a term *t* in HPO is defined as follows:

1 $$ \mathrm{IC}(t)=-\log \frac{N_t}{N} $$

where *N* is the total number of annotated diseases and *N*_*t*_ is the number of diseases annotated by term *t* and all its descendants. When comparing the similarity between two sets of phenotypes, the best pair method just simply counts the number of same terms in both two sets, which does not take the semantic inheritance structure of HPO and the different importance of the terms into consideration.

Thus we propose RelativeBestPair, a new semantic similarity measure based on the information content and the best pair method. Inspired by the idea of information content, we collect diseases annotated by a phenotype t and its descendants to measure the different importance of terms. RealtiveBestPair is described as follows.

A. For a given term *t*, we denote *D*(*t*) as the set of diseases annotated by term *t* and all its descendants and *N*_*t*_ as the size of *D*(*t*). Then, the sccn term *t* is defined as

2 $$ S\left(D\left|t\right.\right)=\left\{\begin{array}{cc}1/{N}_t,& D\in d(t)\\ {}0,& otherwise\end{array}\right. $$

B. Then we can get all the scores of being each disease given each term. For a sets of phenotypes {*t*_*1*_, *t*_*2*_,…, *t*_*n*_} and a disease D_*k*_, the semantic similarity score can be calculated as

3 $$ Sim\left({D}_k\left|{t}_1,{t}_2,\dots, {t}_n\right.\right)={\sum}_{i=1}^n\min \left(\alpha, S\left({D}_k\left|{t}_i\right.\right)\right) $$

where α is a given threshold.

The threshold α is introduced to control the contribution of a single term. If only several diseases are annotated by a single term, then the score of being one of those diseases given this term will be so large that it may dominate the semantic similarity score and ignore the contributions of other terms. For example, we observed a patient with ten terms {*t*_*1*_, *t*_*2*_,…, *t*_*10*_}. If the score of being D_1_ given each of {*t*_*1*_, *t*_*2*_,…, *t*_*9*_} is suitable like 0.005 while the score of being D_2_ given *t*_*10*_ is quite large, for example 0.1, the semantic similarity score between the patient and D_2_ will be larger than that between the patient and D_1_. Thus we use the threshold α to avoid the such extreme cases. Although the choice of α may affect the performance, generally we set it to be 0.01.

**Fig. 2:**
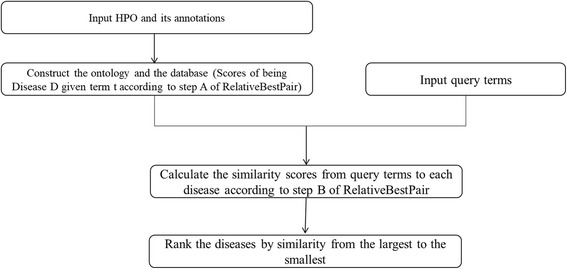
The workflow of disease diagnosis based on RelativeBestPair

Disease diagnosis based on RelaitveBestPair can be summarized as followed (Fig. 2). With the input of HPO and its annotations, the ontology and the database (containing the scores of being each Disease D given each term t using Eq. (2)) are constructed first. Then given a query set of phenotype terms, the similarity scores from query terms to each disease can be calculated with Eq. (3). Finally, diseases are ranked according to these scores from the largest to the smallest.

### Existing semantic similarity measures

We compared the performance of RelativeBestPair with other seven existing approaches summarized in HPOsim [19]. Among those, six approaches are based on information content. The Resnik measure [20], the Lin measure [21], the Jiang-Conrath measure [22], the Relevance measure [23], the information coefficient measure [24] and the graph IC measure [25] define the similarity between two terms as follows:

4 $$ {sim}_{\operatorname{Re} snik}\left({t}_1,{t}_2\right)= IC\left({t}_{MICA}\right) $$

5 $$ {sim}_{Lin}\left({t}_1,{t}_2\right)=\frac{2\times IC\left({t}_{MICA}\right)}{IC\left({t}_1\right)+ IC\left({t}_2\right)} $$

6 $$ {sim}_{JC}\left({t}_1,{t}_2\right)=\frac{1}{\left(1+ IC\left({t}_1\right)+ IC\left({t}_2\right)-2\times IC\left({t}_{MICA}\right)\right)} $$

7 $$ {sim}_{\operatorname{Re}l}\left({t}_1,{t}_2\right)={sim}_{Lin}\left({t}_1,{t}_2\right)\times \left(1-p\left({t}_{MICA}\right)\right) $$

8 $$ {sim}_{IC}\left({t}_1,{t}_2\right)={sim}_{Lin}\left({t}_1,{t}_2\right)\times \left(1-\frac{1}{1+ IC\left({t}_{MICA}\right)}\right) $$

9 $$ {sim}_{GraphIC}\left({t}_1,{t}_2\right)=\frac{\sum_{t\in \left(A\left({t}_1\right)\cap A\left({t}_2\right)\right)} IC(t)}{\sum_{t\in \left(A\left({t}_1\right)\cap A\left({t}_2\right)\right)} IC(t)} $$

Where IC is defined as (1), *t*_*MICA*_ is the most informative common ancestors, *p*(*t*_*MICA*_) is the proportion of diseases annotated by *t*_*MICA*_ and *A*(*t*) is the set of the ancestors of term *t* in HPO.

Besides, the Wang measure [26] is based the structure of ontology. For a given term *t*, *DAG*_*t*_ = (*t*, *T*_*t*_, *E*_*t*_) represents the subgraph made up of term *t* and its ancestors, where *T*_*t*_ is the set of the ancestors of *t* and *E*_*t*_ is the corresponding set of edges is *DAG*_*t*_. In *DAG*_*t*_, *S*_*t*_(*n*) is defined as:

10 $$ \left\{\begin{array}{c}{S}_t(t)=1\\ {}{S}_t(n)=\max \left\{{w}_e\ast {S}_t\left({n}^{\prime}\right)\left|{n}^{\prime}\in \right.\  children\ of(n)\right\} if\ \mathrm{t}\ne \mathrm{n}\end{array}\right. $$

here we choose *w*_*e*_ equal to 0.8. Therefore the similarity between two terms is defined as:

11 $$ {sim}_{Wang}\left({t}_1,{t}_2\right)=\frac{\sum_{t\in {T}_{t1}\cap {Tt}_2}{S}_{t_1}(t)+{S}_{t_2}(t)}{SV\left({t}_1\right)+ SV\left({t}_2\right)} $$

where *SV*(*t*) is the sum of *S*_*t*_(*n*) for *n* in *DAG*_*t*_.

In order to get the similarity between the query set of terms and the set of disease associated terms, we used the one-sided search algorithm as it was showed to be superior to the symmetric version in [7]. The one-sided search algorithm is defined as:

12 $$ {sim}_{one- sided}\left(Q\to D\right)= avg\left[{\sum}_{t_1\in Q}{\max}_{t_2\in D} sim\left({t}_1,{t}_2\right)\right] $$

where *Q* is the set of the query terms (observed phenotypes of the patient), *D* is the set of terms annotated with a given disease, and *sim*(*t*_1_, *t*_2_) can be one of the seven approaches.

Disease diagnosis based the seven semantic similarity measures is quite similar with that based on.

**Fig. 3:**
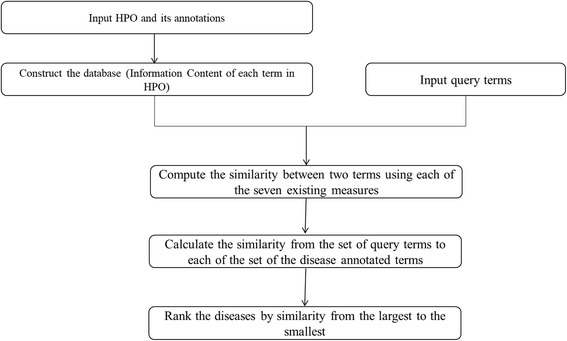
The workflow of disease diagnosis based on the seven existing methods

RelativeBestPair (Fig. 3). Firstly, the ontology and the database (containing information content of each term in HPO) are constructed based on HPO and its annotation files. Secondly, given a query set of phenotype terms, the similarity score from these query terms to each disease are calculated with term-term similarity based on each of the seven methods and then one-sided search algorithm. Finally, diseases are also ranked from the largest score to the smallest score.

### Performance evaluation and generation of simulated patients

Since it is difficult to get clinical features about a large number of patients, we used similar method and same data in [7] to generate simulated patients. In the data used in [7], 44 complex dysmorphology syndromes were identified with detailed frequency of phenotypes. The simulation process is as follows. First, we assigned a disease to each patient. Second, for each phenotype associated with the assigned disease, a random integer between 0 and 100 was generated. If the number was smaller than the relative occurrence in 100 patients (frequency*100), the corresponding phenotype was kept. For each of the 44 diseases, we generated 25 patients with at least three phenotypes. Finally, we got a dataset of 1100 simulated patients. To make the simulation more realistic, three more datasets were also generated just as what was done in [7, 12]. We generated a dataset with ‘noise’ by adding half as many noise terms, unrelated with the underlying disorder, to the present terms, a dataset with ‘imprecision’ by randomly substituting each of the present phenotypes with one of its ancestors in HPO, and also a dataset with both ‘imprecision’ and ‘noise’ by imprecision step first and then noise step. With the four simulated datasets, we evaluated the performance of semantic similarity measure by the ranks of the true disease and adopted the criterion from [12, 19].

## Results

We evaluated the performance of the seven existing approaches and RealtiveBestPair method in the four simulated datasets respectively. We denoted the dataset without noise and imprecision, the dataset with noise and without imprecision, the dataset without noise and with imprecision, and the dataset with both noise and imprecision as “Dataset 1(Noise:-, Imprecision:-)”, “Dataset 2(Noise:+, Imprecision:-)”, “Dataset 3(Noise:-, Imprecision:+)”, and “Dataset 4(Noise:+, Imprecision:+)”. As we moved on from Dataset 1 to Dataset 4, it became more difficult to make the correct diagnosis. It would show us the real abilities of those methods to identify the true underlying disease.

**Fig. 4:**
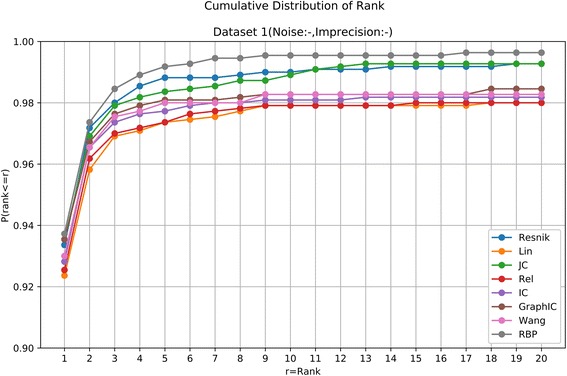
Cumulative Distribution of the rank of the underlying diseases on the simulated dataset without noise and imprecision. The horizontal axis is the threshold for the disease rank. The vertical axis is the corresponding ratio of patients satisfying the ranking threshold

For a given patient, we calculated the similarity score from the patient to each of the 6918 OMIM diseases using one kind of semantic similarity measure, and then rank all the diseases by their similarity scores (from the largest to the smallest). In case that some diseases received the same score, the average rank was returned to make it more reasonable. The results of all the eight methods on the four datasets are shown in Table 1 and Figs. 1, 2, 3 and 4.

**Table 1:** Summary results of different methods on the four simulated datasets

Dataset 1(Noise:-, Imprecision:-)								
	Resnik	Lin	JC	Rel	IC	GraphIC	Wang	RBP
Top 1	1027	1016	1029	1018	1021	1029	1023	1031
Top 5	1087	1071	1082	1071	1075	1079	1078	1091
Top 10	1089	1077	1088	1077	1079	1081	1081	1095
Top 20	1092	1078	1092	1078	1080	1083	1081	1096
Dataset 2(Noise:+, Imprecision:-)								
	Resnik	Lin	JC	Rel	IC	GraphIC	Wang	RBP
Top 1	992	997	1036	996	1006	1031	1001	1030
Top 5	1074	1059	1081	1063	1070	1077	1071	1089
Top 10	1081	1069	1086	1071	1077	1080	1078	1094
Top 20	1087	1074	1089	1076	1078	1083	1079	1095
Dataset 3(Noise:-, Imprecision:+)								
	Resnik	Lin	JC	Rel	IC	GraphIC	Wang	RBP
Top 1	434	243	104	302	336	120	172	438
Top 5	767	502	261	583	603	341	446	765
Top 10	866	613	342	685	707	482	604	863
Top 20	926	714	440	785	797	620	725	926
Dataset 4(Noise:+, Imprecision:+)								
	Resnik	Lin	JC	Rel	IC	GraphIC	Wang	RBP
Top 1	183	130	97	143	162	73	77	370
Top 5	453	327	239	383	406	252	263	694
Top 10	579	452	319	509	533	393	384	786
Top 20	703	570	420	640	657	540	535	860

*Resnik* the Resnik measure, *Lin* the Lin measure, *JC* the Jiang-Conrath measure, *Rel* the Relevance measure, *IC* the information coefficient measure, *GraphIC* the graph IC measure, *Wang* the Wang measure, *RBP* RelativeBestPair method

The seven existing measures are all implemented with one-sided search algorithm. The numbers represent the number of patients in 1100 cases that the true diseases are ranked within top 1, top 5, top 10 or top 20

**Fig. 5:**
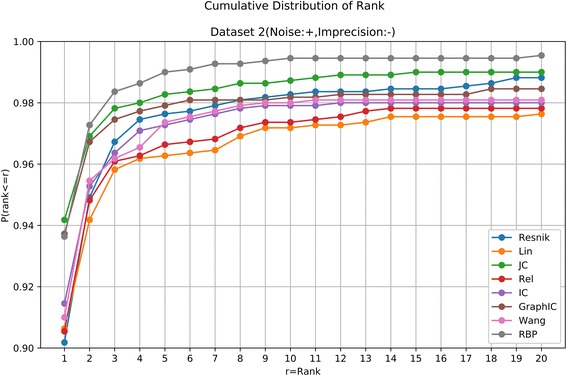
Cumulative Distribution of the rank of the underlying diseases on the simulated dataset with noise and without imprecision. The horizontal axis is the threshold for the disease rank. The vertical axis is the corresponding ratio of patients satisfying the ranking threshold

**Fig. 6:**
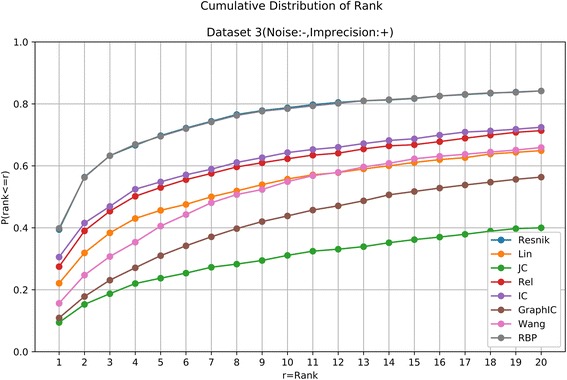
Cumulative Distribution of the rank of the underlying diseases on the simulated dataset without noise and with imprecision. The horizontal axis is the threshold for the disease rank. The vertical axis is the corresponding ratio of patients satisfying the ranking threshold

**Fig. 7:**
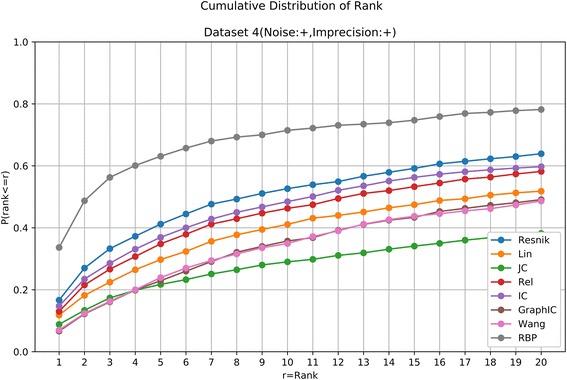
Cumulative Distribution of the rank of the underlying diseases on the simulated dataset with both noise and imprecision. The horizontal axis is the threshold for the disease rank. The vertical axis is the corresponding ratio of patients satisfying the ranking threshold

It can be seen that in the seven existing semantic similarity measures, the Resnik measure has a modest advantage over other six approaches, similar to the results in [7]. The RelativeBestPair method shows the almost the best performance in all four datasets (Table 1). Although in Dataset 1 and Dataset 2, two datasets that do not include “imprecision”, all methods reveal good results by ranking the true diseases as top 1 on over 90% of the patients and within top 20 on over 95% of the patients (Table 1, Figs. 4 and 5), their performances deteriorate with different extents in Dataset 3 and Dataset 4. In Dataset 3 with imprecision, RelativeBestPair method, along with the Resnik measure, tends to be superior with the underlying diseases being ranked within top 1, top 5, top 10, top 20 on 39.82%, 69.55%, 78.45%, 84.18% of the cases for RealtiveBestPair and 39.45%, 69.73%, 78.73%, 84.18% for Resnik (Table 1, Fig. 6). The corresponding percentages using other measures are much smaller. In Dataset 4, a more real situation by both introducing unrelated phenotypic noise and using terms that are more general, RelativeBestPair achieves the best performance among the eight methods (Table 1, Fig. 7). On 33.64% of the patients, their underlying diseases are ranked the highest when applying RelativeBestPair. In comparison, the percentages using Resnik, Lin, Jiang-Conrath, Relevance, information coefficients, Graph IC and Wang measures are only 16.64%, 11.82%, 8.82%, 13%, 14.73%, 6.64% and 7% respectively. Even if a higher rank threshold is employed to give out a candidate list, RelativeBestPair still turns out to be significant better than other methods (Fig. 4). In total, it indicates that RelativeBestPair has the potential to provide a candidate disease/disease list for clinician to improve the diagnosis efficiency as well as accuracy.

## Discussion and conclusion

Recently, the rapid development of sequencing technology makes it possible to get personal genotype data for clinical use, which may be helpful in disease diagnosis. However, the relative high cost and low ability to identify the disease-related causal variants prevent it from being widely used in real cases. While lots of effort and money have been paid to study the relationship between diseases and genetic mutations, to speed up the process of sequencing and to promote the accuracy of sequencing results, in this article we focus on the improvement in the field of phenotypic diagnosis. Compared with genotypic data, it is much easier to get phenotypic data from patients. With the construction and development of the Human Phenotype Ontology and the enrichment and completeness of disease-phenotype annotations, the observed phenotypes of a particular patient can provide more information about the underlying disease he/she might suffer.

Here we proposed a novel method called RelativeBestPair to measure the semantic similarity from a given set of phenotypes to a disease. Different from those existing approaches that calculate the similarity from the query set to a certain disease based on term-term comparison, we directly define the contribution of one phenotype term to the certain disease. To evaluate the performance of RelativeBestPair and seven existing methods, we adopted the procedure similar to that in [7, 12] to generate four kinds of simulated patients from the easiest situation to the most difficult situation. In order to be adapted to the scenario of disease diagnosis, the one-sided search algorithm, which showed better performance than symmetric version in [7], was chosen for the seven existing methods. The results on the simulated datasets demonstrated that RelativeBestPair outperformed other methods in all situations especially when “noise” and “imprecision” were added, typical in the clinical setting.

Despite the well performance in simulation, there still remains much for RelativeBestPair to take into consideration. Firstly, the optimal value for α requires further discussion. The introduction of threshold α played a key role in the performance of RelativeBestPair since we found poor results when the threshold α was not employed. Therefore, the choice of threshold α would substantially affect the performance. Other than 0.01, we also tested other values for α including 0.001–0.005, 0.015, 0.02, 0.025 and 0.03. Although those results showed some minor difference (data not shown), considering the fact that on average one term annotates about 150 diseases which indicates that average score of being the given disease is 1/150 ≈ 0.0067, empirically the choice of 0.01 for α might be enough to make sure that the contribution of one single term won’t be too large. Other choices are also welcomed as long as α is neither too big nor too small. Secondly, unlike the seven existing approaches, RelativeBestPair cannot be used to compute the similarity between two phenotype terms. The usage of RelativeBestPair might be limited in disease diagnosis and its expansion to other biomedical ontologies and other usages may be uncertain. Finally, without thousands of real cases, the true ability of RelativeBestPair as well as other semantic similarity measures in disease diagnosis is still unknown. As mentioned before, all the simulations are based on 44 complex diseases with detailed frequencies of phenotypes [7]. Then, we cannot assert the performance in any cases. However, from the simulation results, RelativeBestPair might have a large potential to identity the true underlying diseases of patients.

In conclusion, we have presented a new method, RelativeBestPair, that calculates the semantic similarity from the given query terms to each disease. Our method has the advantage of pay special attention to the fields of disease diagnosis. This approach can be applied to the real clinical setting by providing clinicians with a candidate disease list. We have shown that RelativeBestPair achieved a better performance of identifying the true disease as top-ranked diseases against other methods in four simulated dataset, mimic to the real cases.
